# Ecological adaptation in European eels is based on phenotypic plasticity

**DOI:** 10.1073/pnas.2022620118

**Published:** 2021-01-21

**Authors:** Erik D. Enbody, Mats E. Pettersson, C. Grace Sprehn, Stefan Palm, Håkan Wickström, Leif Andersson

**Affiliations:** ^a^Department of Medical Biochemistry and Microbiology, Uppsala University, 751 23 Uppsala, Sweden;; ^b^Department of Aquatic Resources, Institute of Freshwater Research, Swedish University of Agricultural Sciences, 178 93 Drottningholm, Sweden;; ^c^Department of Veterinary Integrative Biosciences, Texas A&M University, College Station, TX 77843;; ^d^Department of Animal Breeding and Genetics, Swedish University of Agricultural Sciences, 750 07 Uppsala, Sweden

**Keywords:** adaptation, panmixia, plasticity, genome biology

## Abstract

The relative role of genetic adaptation and phenotypic plasticity is of fundamental importance in evolutionary ecology [M. J. West-Eberhard, *Proc. Natl. Acad. Sci. U.S.A.* 102 (suppl. 1), 6543–6549 (2005)]. European eels have a complex life cycle, including transitions between life stages across ecological conditions in the Sargasso Sea, where spawning occurs, and those in brackish and freshwater bodies from northern Europe to northern Africa. Whether continental eel populations consist of locally adapted and genetically distinct populations or comprise a single panmictic population has received conflicting support. Here we use whole-genome sequencing and show that European eels belong to one panmictic population. A complete lack of geographical genetic differentiation is demonstrated. We postulate that this is possible because the most critical life stages—spawning and embryonic development—take place under near-identical conditions in the Sargasso Sea. We further show that within-generation selection, which has recently been proposed as a mechanism for genetic adaptation in eels, can only marginally change allele frequencies between cohorts of eels from different geographic regions. Our results strongly indicate plasticity as the predominant mechanism for how eels respond to diverse environmental conditions during postlarval stages, ultimately solving a long-standing question for a classically enigmatic species.

## Significance

Populations within a species may experience genetic adaptation to local environmental conditions while individuals may tolerate variation in environmental conditions due to phenotypic plasticity. European eels reproduce in the Sargasso Sea but spend most of their adult life in highly diverse environmental conditions from northern Europe to North Africa. An unresolved question is whether this species constitutes a single panmictic population or if locally adapted subpopulations occur. Here we conclude, based on whole-genome sequencing, that there is a complete absence of genetic differentiation among eels from different parts of Europe and North Africa. Thus, we postulate that European eels must respond to the extremely diverse ecological conditions they inhabit during the postlarval stages by phenotypic plasticity.

How species adapt to the diverse environmental conditions that they experience through their life is fundamental to understanding evolutionary processes. Species that occur across extreme environmental gradients must respond to a diverse range of conditions. This can be accomplished by individual-level phenotypic plasticity, meaning that individuals adjust their physiology to the prevailing environmental conditions (1), and by local genetic adaptation that may lead to reproductively isolated subpopulations.

The European eel (*Anguilla anguilla*) provides a fascinating example. Adult eels are mostly found in freshwater bodies and brackish coastal areas from North Africa in the south to the North Cape in the north (well above the polar circle), from the Azores in the west to the Black Sea in the east. The spawning grounds long remained a mystery, but 100 y ago larvae were discovered in the Sargasso Sea, ∼7,000 km away from the mainland (2), and subsequent research has only recently begun to reveal the complexity of their spawning migration as maturing adults back to the Sargasso Sea (3–5). All European eels reproduce in the Sargasso Sea and the offspring drift passively, as leptocephali larvae, on oceanic currents toward the European continent. Consequently, which geographic region across Europe and North Africa eels inhabit as adults appears to be driven largely by a stochastic process. These observations are consistent with a single panmictic population and would preclude genetic adaptation to local conditions in Europe and North Africa. However, eel reproduction in the Sargasso Sea, which is 2,000 km wide, does not exclude the possibility of genetically differentiated subpopulations with distinct spawning areas or timing which may impact the likelihood of which geographic region the larvae reach after their trans-Atlantic migration. In fact, European eels and American eels (*Anguilla rostrata*), estimated to have split from a common ancestor around 3.75 million y ago (6), reproduce in parapatry in the Sargasso Sea but still maintain reproductive isolation with a low rate of hybridization (7, 8). Evaluating the hypothesis that the European eel consists of a single panmictic population is central to understanding eel ecology and evolution, and how eel populations may be affected by global change and other environmental threats (9, 10).

Previous studies on the European eel using low-density marker sets (6, 11–15) or reduced representation sequencing (15) found little to no differentiation between geographic areas, consistent with a single panmictic population. However, low genetic differentiation at selectively neutral markers is a common observation in marine species with large geographical ranges and gene flow between subpopulations (16, 17), but does not necessarily capture patterns of local adaptation. For example, an early study based on 13 allozyme loci failed to identify genetic differentiation among Atlantic and Baltic herring (*Clupea harengus*) from diverse ecological conditions, and a single panmictic population could not be excluded (18). In sharp contrast, whole-genome sequencing revealed strong genetic differentiation between ecotypes of herring for a few percent of all genes (19–21). Thus, a lack of genetic differentiation even at many loci does not exclude the possibility that the European eel is divided into partially reproductively isolated subpopulations, genetically adapted to the diverse ecological conditions that individuals are exposed to during postlarval stages. Whole-genome data are required to evaluate the possibility that European eel populations are structured into subpopulations showing genetic differentiation.

**Fig. 1.**
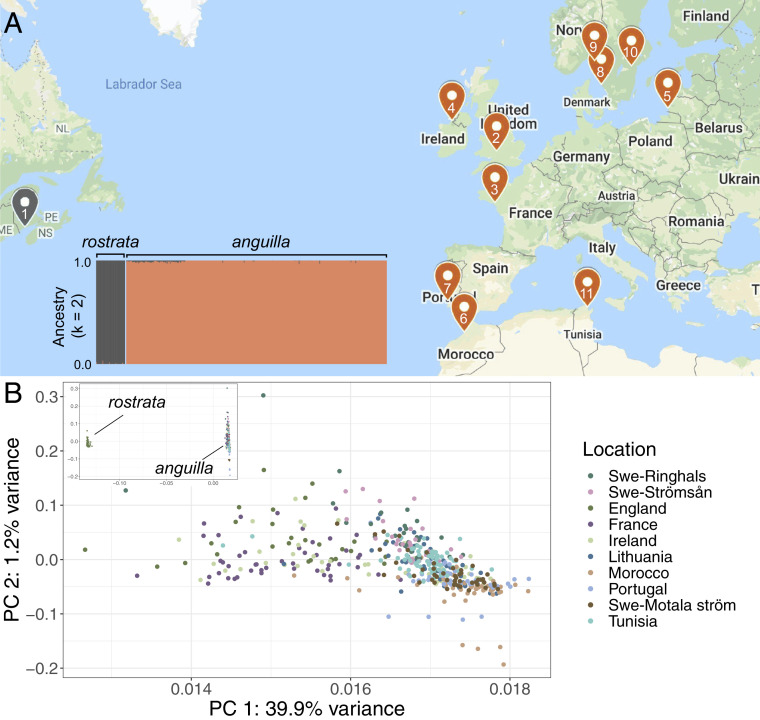
Sample overview and population structure analysis. (*A*) Sample locations (*SI Appendix*, Table S1) with NGSadmix (23) ancestry proportions for American (*A. rostrata*) and European (*A. anguilla*) eel samples (*inset*). *K* = 2 is best supported; see *SI Appendix*, Fig. S1 for additional analyses. (*B*) Principal-component analysis for whole-genome SNPs generated in PCAngsd (25), with points colored by sampling locality. PC 1 variance (39.9%) is driven by species divergence and *A. rostrata* ancestry in *A. anguilla* samples.

A high-quality reference genome for the European eel has recently been released by the Vertebrate Genomes Project (22). Here we used this assembly and low-coverage, individual whole-genome sequencing of 445 individuals from 10 geographic samples (median sequence coverage 1.4) covering most of the species range, stretching from Sweden to Ireland to Tunisia (Fig. 1*A* and *SI Appendix*, Table S1). We also include a sample of American eel (*n* = 49) as an outgroup. Our study scales considerably over existing genomics research on the European eel, both in number of individuals and number of loci sequenced (15). The sample design and sample size were selected to critically evaluate the presence of any genetic differentiation (due to drift or selection) between individuals from different geographic regions. We report a complete lack of genomic regions with significant differentiation between geographically separated samples.

## European Eels Constitute a Panmictic Population

Our data show that the European eel is a highly polymorphic species with an average nucleotide diversity of 1.4%, which is 14-fold higher than in humans. We assessed population structure in European and American eels using genotype likelihoods with the NGSadmix (23) module from the ANGSD package (24). Structure plots clearly demonstrated a complete lack of population differentiation among the European samples, and the two clusters correspond to the division between species (Fig. 1*A* and *SI Appendix*, Fig. S1). A few individuals from the English, Irish, and French samples have a small, but detectable, contribution of American genomic composition (maximum 3%), which could indicate ongoing gene flow at a low rate, consistent with the sporadic occurrence of F_1_ hybrids reported elsewhere (7, 8). A principal-component analysis (PCA) based on the entire genome supports a lack of discernible population structure and the distribution of European eel samples along PC 1 corresponds to the small contribution *of A. rostrata* ancestry (not to genetic structure within *A. anguilla*; Fig. 1*B*).

**Fig. 2.**
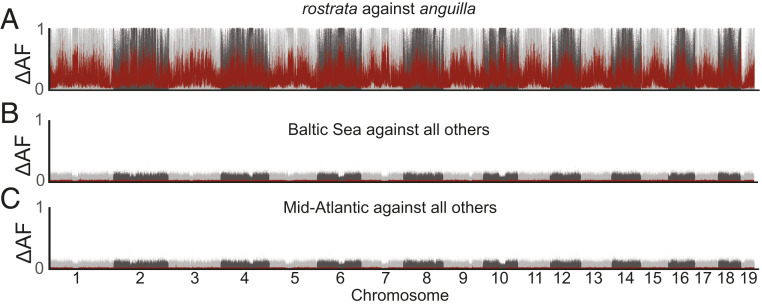
Population contrasts. (*A*) Delta allele frequencies (ΔAFs) between American eel (*A. rostrata*) and all European eel (*A. anguilla*) samples. Gray dots are single SNPs, while the red line is the 100-SNP rolling average. (*B*) Samples from the Baltic Sea, or fresh waters connected to the Baltic Sea, versus all other European eel samples. (*C*) Mid-Atlantic samples (England, Ireland, France) versus all other European eel samples.

In order to evaluate if genetic differentiation is restricted to a small number of single-nucleotide polymorphisms (SNPs) or haplotypes, we scanned the genome for regions associated with location or environmental conditions using per-SNP delta allele frequency contrasts, based on SNPs with at least 5% minor allele frequency across the entire sample set. First, we compared European and American eels and demonstrated that this approach reveals genome-wide high divergence, as expected, with hundreds of thousands of fixed differences all over the genome (Fig. 2*A*). In sharp contrast, comparisons between groups of European eels from different geographic regions are devoid of signals (Fig. 2 *B* and *C*), with only the “mid-Atlantic” grouping (i.e., English, Irish, and French samples) mentioned above showing a weak but consistent signal covering roughly 6 kb around 81.2 Mbp on chromosome 1 (*SI Appendix*, Fig. S2). This signal covers the first three exons of *LOC118232784* (myelin-associated glycoprotein-like) and includes one missense mutation (Chr1:81,197,679 T/A). In this specific region, shared polymorphisms are segregating in both European and American eels and the “mid-Atlantic” samples are more similar to the Canadian sample than to other European samples (*SI Appendix*, Fig. S2). This suggests some ongoing gene flow, as discussed above, or a balanced polymorphism. This SNP-based approach could have missed population-specific small insertions and deletions or structural variants, but it is unlikely that population differentiation is caused exclusively by such variants and that these variants do not show linkage disequilibrium to SNPs in the near vicinity.

**Fig. 3.**
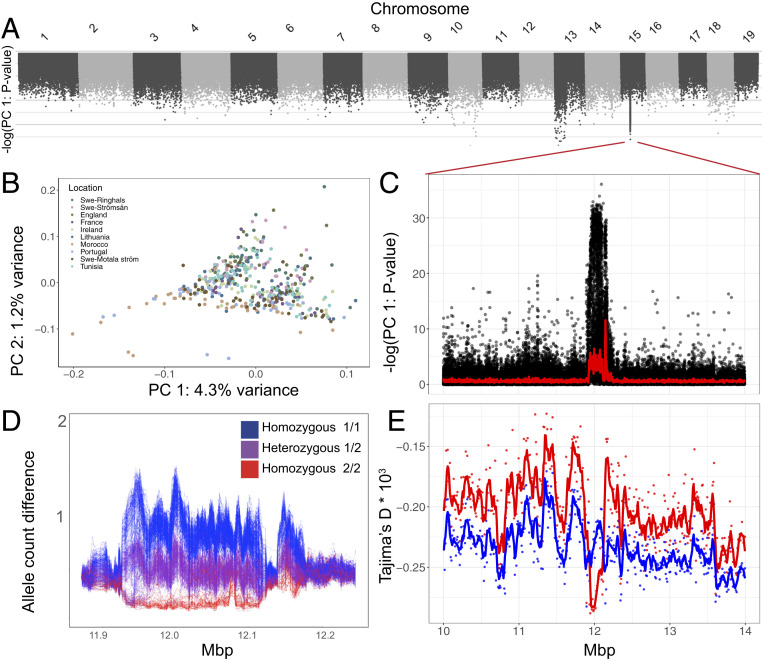
Whole-genome principal-component analysis. (*A*) *P* values for SNPs contributing to PC 1 across the genome. (*B*–*E*) In-depth analysis of the signal on chromosome 15. (*B*) PC 1 versus PC 2. (*C*) A reversed zoom-in from *A* on the peak around 12 Mbp on chromosome 15 and a red line showing the rolling average per 50 SNPs. (*D*) Allele count differences between individual samples based on genotype likelihoods across a 100-SNP sliding window. Individuals are colored according to their estimated genotype. (*E*) Tajima’s *D* for the two groups of estimated homozygotes across the same region as in *C*.

Lastly, we used a data-driven and population-blind approach to identify putative targets of selection that could have gone undetected based on our population classifications. We searched for SNPs whose distribution exceeds what is expected under neutrality along the first principal component using PCAngsd (25). We detected two major outlier regions, on chromosomes 13 and 15 (Fig. 3*A*), which segregate at intermediate frequency in every sampling locality. In other words, neither genomic region is associated with differentiation among localities. The signal on chromosome 13 covers a 6-Mbp region where around 10% of the sampled individuals carry rare alleles in strong linkage disequilibrium for a subset of markers (*SI Appendix*, Fig. S3). We explored if this pattern could be consistent with a sex chromosome [undescribed in eels, believed to have a nongenetic sex determination mechanism (26)], but analysis of published transcriptome data is inconsistent with this hypothesis (*SI Appendix*, *Text*).

## Large Haplotype Introgression

The signal on chromosome 15 is strong enough to affect the PCA for the entire chromosome (Fig. 3*B*), although it only spans ∼200 kb, from 11.95 to 12.15 Mbp (Fig. 3*C*). We extracted the markers most likely to contribute to PC 1 in this region and calculated a kinship matrix based on the individual genotype likelihoods for those SNPs. Using this method, 442 out of 445 individuals could be placed into three discrete groups (*SI Appendix*, Fig. S4). Comparing the estimated allele counts for each individual shows that this pattern is consistent across the region for the vast majority of samples but then quickly deteriorates in either direction (Fig. 3*D*), a pattern consistent with an inversion or a region of otherwise reduced recombination. The less common version (haplotype 2) is also the younger, as it shows a more negative Tajima’s *D* than haplotype 1 (Fig. 3*E*). This is mostly driven by a lack of within-group diversity among haplotype 2 homozygotes, which is not seen to the same extent among haplotype 1 homozygotes (*SI Appendix*, Fig. S5).

The frequency of haplotype 2 has no significant association with any subpopulation (per-population frequency ranges from 22 to 37%, overall frequency 29%, *P* = 0.96 [χ^2^ test, degrees of freedom = 9]), and the per-population frequencies do not covary with any obvious environmental gradient. Given the comparatively high frequency of the younger version, it is unlikely to be a neutral polymorphism. Furthermore, the frequency of haplotype 2 is close to 100% in the American eel sample (*SI Appendix*, Fig. S6 *A* and *B*) and the locus displays a drastic loss of diversity (*SI Appendix*, Fig. S6*C*), a feature not found in European samples (*SI Appendix*, Fig. S6*D*). Thus, it is likely that this haplotype has undergone a selective sweep in the American eel, and introgressed into European eels. Based on the absence of genetic differentiation among geographic regions sampled in this study, we predict that the variant is possibly associated with selection at the egg or larval stages, related to the large range of spawning areas within the Sargasso Sea. The annotated genes within the putative inversion are discussed in *SI Appendix*, *Text*.

## Discussion

The question of whether or not the European eel is a truly panmictic species has been repeatedly studied and debated over the years (13, 15, 27, 28). Our results, based on whole-genome sequencing, provide ultimate and conclusive evidence that it should be considered as one single panmictic population. Unlike all other previous studies of fish species living across major salinity and temperature gradients (e.g., refs. 17, 21, and 29), we find no evidence for local genetic adaptation in the form of genome-wide or narrow regions of selection by examining geographic allele frequency differentiation. Eels are exposed to extreme differences in ecological conditions due to their presence in marine, brackish, and freshwater environments from northernmost Europe to northern Africa. Our principal-component analysis should have detected genetic differentiation even if ecological adaptation had a highly polygenic background with only small shifts in allele frequencies at many loci. In fact, our agnostic PCA-based selection scan convincingly demonstrates a putative inversion that has adaptively introgressed from the American eel, which highlights the power of our dataset to detect signatures of potential local adaptation.

The observation of American eel ancestry in most English, Irish, and French samples is a small, but significant, deviation from the expected pattern under complete panmixia. The observed westerly distribution of *A. rostrata* ancestry in Europe mirrors the westerly distribution of F_1_ hybrids found in the Sargasso Sea [and dispersed F_1_ in Iceland (30)]. As a consequence, this small (3%) introgression of American germplasm, that seemingly reflects multiple generations of backcrossing with pure *A. anguilla*, is more likely to reflect larval dispersal mechanisms than selection for genome-wide *A. rostrata* SNPs in mid-Atlantic populations.

How is it possible that the European eel, a poikilothermic organism, can adjust its metabolism to thrive in widely different environmental conditions from northern Europe, where the water temperature in lakes during winter is +4 °C, to northern Africa, where water temperatures in freshwater bodies can be +30 °C or higher, without any significant genetic differentiation in the genome? The situation is in sharp contrast to the Atlantic herring which shows striking genetic differentiation at nonneutral loci between geographic regions with less extreme environmental differences (21). The crucial difference between these two species is that in the Atlantic, herring spawning and embryonic development take place under diverse environmental conditions whereas all European eels are thought to spawn in the Sargasso Sea. The most sensitive period of life for a fish is during embryonic development and early larval life (31). In contrast to a previous reduced representation sequencing study (15), we did not find evidence for within-generation selection affecting the earliest life stages to contrasting continental environments. Within-generation selection will cause considerable mortality each generation, unless it affects a limited number of loci and causes too small shifts in allele frequencies to be detected with the sample sizes used in the present study. For example, a mortality of 84% is required to cause only a 5% shift in allele frequency at 10 codominant loci in one geographic region, and only 1 out of 10^8^ individuals survives after such shifts in allele frequencies at 100 loci (*SI Appendix*, *Text*). A 5% allele frequency shift at 100 loci would still be small compared with genetic differentiation, for instance in the Atlantic herring, where ecological adaptation to the brackish Baltic Sea involves strong allele frequency shifts at hundreds of loci, many approaching fixation for different alleles (20, 21). We therefore conclude that within-generation selection, as recently proposed for both the European and American eel (15, 27), can only have marginal effects on local allele frequencies, and postulate that eels tolerate diverse ecological conditions largely based on plasticity.

Our results suggest that constraints imposed by spawning and embryo development in the Sargasso Sea apparently preclude reproductive isolation between continental subpopulations. This implies that during millions of years there has been strong selection for plasticity at the adult stage, which allows eels to inhabit large environmental gradients across Europe and North Africa. Eels exhibit plasticity while migrating between oceanic environments, to brackish and fresh water and back to oceanic water again. Eels have even been known to migrate short distances on land (32), and can survive in small man-made wells for decades. The molecular mechanisms underlying this plasticity have not been explored in detail but may involve epigenetic regulation (DNA methylation and histone modifications) as well as protein stabilization mechanisms (e.g., chaperones) over a wide temperature range. These may be studied after exposing cohorts of eels to different relevant environmental variables such as temperature.

Recruitment of young European eels has declined substantially across Europe in the last century (9, 10). Our results imply that this species constitutes a single breeding population. Clearly, international cooperation is essential to avoid a further reduction in population size, as overfishing or disturbed environmental conditions in one geographic region may affect glass eel recruitment across the entire species distribution.

## Methods

### Sample Collection.

The samples used in this study were selected from the sample collection previously used by Dannewitz et al. (33) combined with samples collected at the Swedish west coast in 2019 (*SI Appendix*, Table S1). The aim was to get a good representation across Europe and North Africa including an outgroup sample from Canada (American eel). The majority of samples (8 out of 11) constituted glass eels of unknown sex.

### Whole-Genome Sequencing.

DNA was extracted using Qiagen Blood & Tissue 96 Kits. DNA was quantified using the Qubit and Tecan plate reader, and diluted to 10 ng/μL for library preparation. We created custom Tn5-based libraries (34) for individuals from 11 populations targeting a fragment size of 350 bp. Individually barcoded libraries were pooled and a TapeStation D1000 was used to visualize library size. Pools were sequenced on two lanes of Illumina NovaSeq S4. One lane was run on a single Illumina MiniSeq flow cell to check sample coverage prior to sequencing on the NovaSeq.

### Mapping.

The Illumina short reads were mapped to the primary fAngAng1 assembly (GCA_013347855.1), which was produced as part of the Vertebrate Genomes Project (22). Mapping was done using “bwa mem -M” v0.7.17 (35). The resulting alignments were sorted using Samtools v1.10 (http://www.htslib.org/) and finally processed with MarkDuplicates from PicardTools v1.92 (https://broadinstitute.github.io/picard/).

### Genotype Likelihood Estimation and Minor Allele Frequency Cutoff.

We used ANGSD v0.933 (24) to estimate genotype likelihoods for all analyses, because our low sequencing coverage approach prohibits calling genotypes. Instead, we used genotype likelihoods to estimate population allele frequencies (based on 28 to 67 individuals per location; *SI Appendix*, Table S1). For all runs, we used the following parameters: “-uniqueOnly 1 -remove_bads 1 -only_proper_pairs 0 -trim 0 -GL 2.” Depending on the analysis, we used the “–doMajorMinor 4 –minMaf 0.05 or 0.1” combination to generate lists of positions with minor allele frequency (MAF) >5 or >10%, respectively, for all 19 chromosomes. These were then supplied as the “-sites” arguments for PCA, genotype likelihood, and population-wise allele frequency calculations, while diversity and Tajima’s *D* calculation used all observed positions.

### Population Structure.

We ran NGSadmix using a downsampled list of 2 million sites (using the MAF > 0.1 filter) and supplying the -SNP_pval 1e-6 to ANGSD to include only high-confidence variants. We ran NGSadmix with both *k* = 2 and *k* = 3 and visual inspection of the results clearly excluded population structure within the run with *k* = 3 (*SI Appendix*, Fig. S1). Furthermore, we calculated individual admixture proportions using PCAngsd v0.985 (25) which empirically sets *k* based on PC loadings and resulted in calculating admixture with *k* = 2. The results for admixture in PCAngsd and NGSadmix were qualitatively the same, and only the results for NGSadmix are presented for population structure. Four individuals were removed from population structure visualizations (AMAR7, ATU6, APM45, AMAR10) due to excessively low sequencing coverage after inspecting coverage estimates produced using the software Indexcov (36) and eel bam file indexes.

### Population Contrasts.

The frequencies for each component of the contrast were calculated using ANGSD (24) “–doMajorMinor 4” on the sites that exceeded the study-wide 5% MAF threshold (see above). This implies we are unable to detect differentiation that occurs exclusively in rare SNPs with a frequency below this threshold.

### PCA-Based Selection Scan.

We used the -selection flag in PCAngsd to calculate a selection statistic per SNP as described in ref. 37 for each chromosome individually, using a subset of SNPs filtered with the MAF >0.1 cutoff. This analysis was run with American eel (*A. rostrata*) removed. The test statistic follows a χ^2^ distribution with 1 degree of freedom and per-SNP probabilities were calculated using the pchisq function (two-tailed mode; i.e., lower.tail = TRUE) for the first principal component in R (v3.6.1) (38). The covariance matrix output with PCAngsd was used to visualize principal components and eigenvectors were calculated for the covariance matrix using the eigen function in R (v3.6.1) (38).

### Nucleotide Diversity.

We calculated thetas using allele frequencies calculated from a callset that was not filtered for MAF cutoffs. Any MAF cutoff will bias site frequency spectrum-based diversity estimates. We first generated a consensus fasta file of *A. rostrata* samples using the angsd -getfasta 2 command to polarize *A. anguilla* alleles. We next calculated sample allele frequencies per sampling locality using the following command:

$ANGSD_PATH/angsd -bam ${POPULATION}_bamlist.txt -doSaf 1 -anc american_ell.fasta -GL 2 -P 8 -out $POPULATION -doCounts 1 -setMinDepth 15 -setMaxDepth 1000 -setMinDepthInd 0.25 -minMapQ 1 -minQ 20 -remove_bads 1 -uniqueOnly 1 -dumpCounts 2 -doMajorMinor 5 -doMaf 2

Our filters were selected to remove extreme outliers in sequencing depth and to remove spurious alignments and low-quality sites. We next used the ANGSD realSFS command to generate the unfolded site frequency spectrum, which was used as a prior for calculating diversity statistics with the saf2theta command (24). Pairwise nucleotide diversity was calculated in 5-kb nonoverlapping windows and averaged per population, dividing by the number of sites per window to recover an unbiased estimate of diversity.

## Supplementary Material

Supplementary File

## Data Availability

All DNA-sequencing data from this study are available through the National Center for Biotechnology Information Sequence Read Archive (BioProject ID code PRJNA668259). All code used to analyze sequence data are available at https://github.com/erikenbody/eel_code_pub.
