# Supplementary material for: Ecological adaptation in European eels is based on phenotypic plasticity
Source: Proc Natl Acad Sci U S A. 2021 Jan 21;118(4):e2022620118. doi: 10.1073/pnas.2022620118 (PMC7848574; doi:10.1073/pnas.2022620118)
Supplement: Supplementary File [file pnas.2022620118.sapp.pdf]

Supplementary Information Appendix

Fig. S1. Admixture analysis using NGSAdmix with parameter values of *k*=2 (A) or *k*=3 (B). One bar refers to a single individual's proportional ancestry and sampling localities are demarcated along the x-axis. Results for *k*= 2 are the best supported for this dataset and clearly separate *A. anguilla* (orange) and *A. rostrata* (blue) species. (C) Admixture results with *A. rostrata* removed for *k*=2. In (D) we used box-and-whiskers plots to illustrate the minor ancestry proportion for each sampling locality (note the scale of the y-axis). Orange refers to proportion of ancestry of *A. rostrata* in each individual *A. anguilla* and blue refers to the proportion of *A. anguilla* ancestry in *A. rostrata* samples. The maximum *A. rostrata* ancestry in an *A. anguilla* individual is 3.6% in a Swedish sample (ASRI). AST=Strömsån, Sweden; SMS= Motala ström, Sweden; ASRI=Ringhals, Sweden.

Fig. S2. Genetic variation at chromosome 1:81 Mbp. (A) Delta allele frequency (DAF) between Mid Atlantic samples (Ireland, England, and France) and the remaining *A. anguilla* samples. Blue dots are SNPs shown in the heatmap in panel B, and the red dot indicates a missense mutation. (B) Per-population frequencies for differentiated SNPs in the region.

Fig. S3. Genetic variation at chromosome 13:1-7 Mbp. (A) Heatmap where individual *A. anguilla* samples have been clustered based on genotype similarity for >2000 SNPs with high contribution to PC 1 for chromosome 13. (B) Plot of allele frequencies among group 1 individuals on the x-axis vs. group 2 individuals on the y-axis (groupings as indicated in panel A). All frequencies have been transformed to show the minor allele in the Type 1 group. Purple dots represent the SNPs used to construct the heatmap in panel A, grey dots are a set of approximately 10^4 randomly selected background SNPs, and the diagonal line shows equal frequencies in the two groups. As can be seen, the diagnostic SNPs are characterized by being fixed in the majority Type 1 group, while being close to 50% in the Type 2 group suggesting that these are heterozygous at these loci. In contrast, the background loci show very similar allele frequencies in the two groups. (C) Delta allele frequency between groups is shown on the lower left-hand side, with markers colored as in panel B. The dark (group 1) and light (group 2) grey dots are estimated depths of coverage in the two groups. The red line indicates annotated genes over the region containing divergent SNPs. SNPs are colored as in panel B. It is noteworthy that both diagnostic (purple) and background SNPs occur along the entire region showing this signal.

Fig. S4. Genetic polymorphism at chromosome 15:12 Mbp region. (**A**) Heatmap where the individual *A. anguilla* samples have been clustered based on genotype similarity for >700 SNPs with high contribution to PC 1 for chromosome 15. (**B**) Delta allele frequency (DAF) between groups. Blue dots are SNPs used for the heatmap in panel A, and the red dots indicate missense mutations.

Fig. S5. Characterization of putative inversion on chromosome 15:12 Mbp region. (**A**) Probability of finding different SNP alleles between the two haplotypes in haplotype 1 homozygotes and heterozygotes. (**B**) Same information as in panel A but for haplotype 2.

Fig. S6. Further characterization of the putative inversion on chromosome 15:12 Mbp region. (**A**) Alternative (i.e. non-reference) allele frequencies for diagnostic SNPs of all 11 populations. Clearly, all samples are very similar except for the Canadian *A. rostrata* sample (ACA, bolded in the legend, turquoise diamonds); sample abbreviations are as described in Table S1. (**B**) Mean allele frequencies for homozygotes of the two haplotypes (as estimated from individual genotype likelihoods), as well as an indication of whether or not the reference corresponds to the most common (i.e. "European") haplotype or not. (A value of 1 means they are the same, 0 means they differ). The pattern indicates that the reference individual was a heterozygote, and that the assembly has undergone a haplotype-switch in the repeat block located around 12.13 Mbp. Before the switch, the assembly is "American", after it is "European." (**C**) Allele frequencies for all SNPs in the region for the *A. rostrata* eel sample. (**D**) Allele frequencies for all SNPs in the region for the Ringhals samples (Sweden) illustrating higher nucleotide diversity in this region among *A. anguilla* samples.

Table S1. Sample metadata for all eleven localities included in this study

Supplementary Text

Literature cited in Supplementary Information Appendix

1. J. Dannewitz, *et al.*, Panmixia in the European eel: a matter of time. *Proc. R. Soc. B Biol. Sci.* **272**, 1129–1137 (2005).
2. A. M. Churcher, P. C. Hubbard, J. P. Marques, A. V. M. Canário, M. Huertas, Deep sequencing of the olfactory epithelium reveals specific chemosensory receptors are

expressed at sexual maturity in the European eel *Anguilla anguilla*. *Mol. Ecol.* **24**, 822–834 (2015).

3. A. Dobin, *et al.*, STAR: Ultrafast universal RNA-seq aligner. *Bioinformatics* **29**, 15–21 (2013).

4. A. McKenna, *et al.*, The genome analysis toolkit: A MapReduce framework for analyzing next-generation DNA sequencing data. *Genome Res.* **20**, 1297–1303 (2010).

5. D. Freed, R. Aldana, J. A. Weber, J. S. Edwards, The Sentieon Genomics Tools - A fast and accurate solution to variant calling from next-generation sequence data. *bioRxiv*, 115717 (2017).

6. U. H. Wiberg, Sex determination in the European eel (*Anguilla anguilla*, L.). A hypothesis based on cytogenetic results, correlated with the findings of skewed sex ratios in eel culture ponds. *Cytogenet Cell Genet.* **36**, 589–598 (1983).

7. S. Bonhommeau, *et al.*, Estimates of the mortality and the duration of the trans-Atlantic migration of European eel *Anguilla anguilla* leptocephali using a particle tracking model. *J. Fish Biol.* **74**, 1891–1914 (2009).

8. C. M. F. Durif, *et al.*, Age of European silver eels during a period of declining abundance in Norway. *Ecol. Evol.* **10**, 4801–4815 (2020)

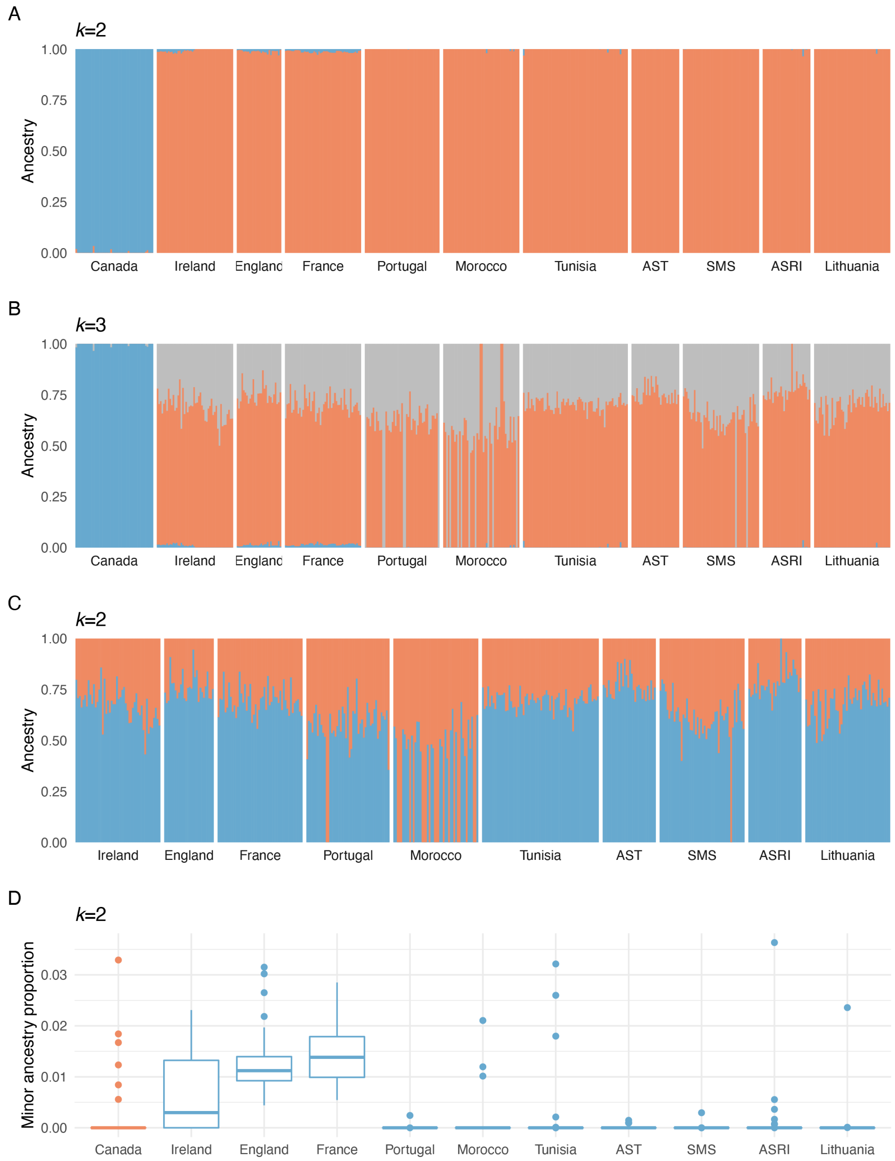

Fig. S1. Admixture analysis using NGSAdmix with parameter values of *k*=2 (A) or *k*=3 (B). One bar refers to a single individual's proportional ancestry and sampling localities are demarcated along the x-axis. Results for *k*= 2 are the best supported for this dataset and clearly separate *A. anguilla* (orange) and *A. rostrata* (blue) species. (C) Admixture results with *A. rostrata* removed for *k*=2. In (D) we used box-and-whiskers plots to illustrate the minor ancestry proportion for each sampling locality (note the scale of the y-axis). Orange refers to proportion of ancestry of *A. rostrata* in each individual *A. anguilla* and blue refers to the proportion of *A. anguilla* ancestry in *A. rostrata* samples. The maximum *A. rostrata* ancestry in an *A. anguilla* individual is 3.6% in a Swedish sample (ASRI). AST=Strömsån, Sweden; SMS= Motala ström, Sweden; ASRI=Ringhals, Sweden.

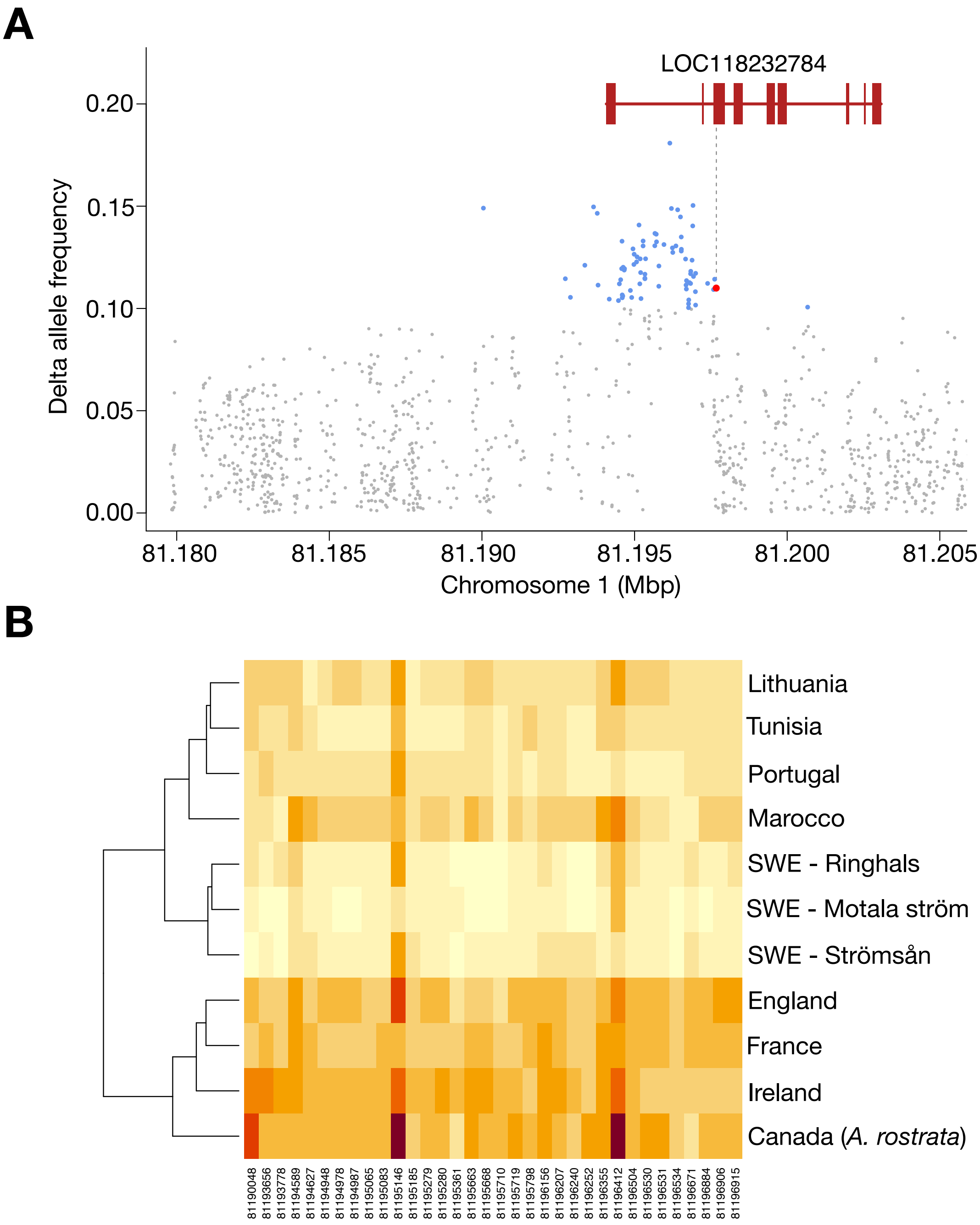

Fig. S2. Genetic variation at chromosome 1:81 Mbp. (A) Delta allele frequency (DAF) between Mid Atlantic samples (Ireland, England, and France) and the remaining *A. anguilla* samples. Blue dots are SNPs shown in the heatmap in panel B, and the red dot indicates a missense mutation. (B) Per-population frequencies for differentiated SNPs in the region.

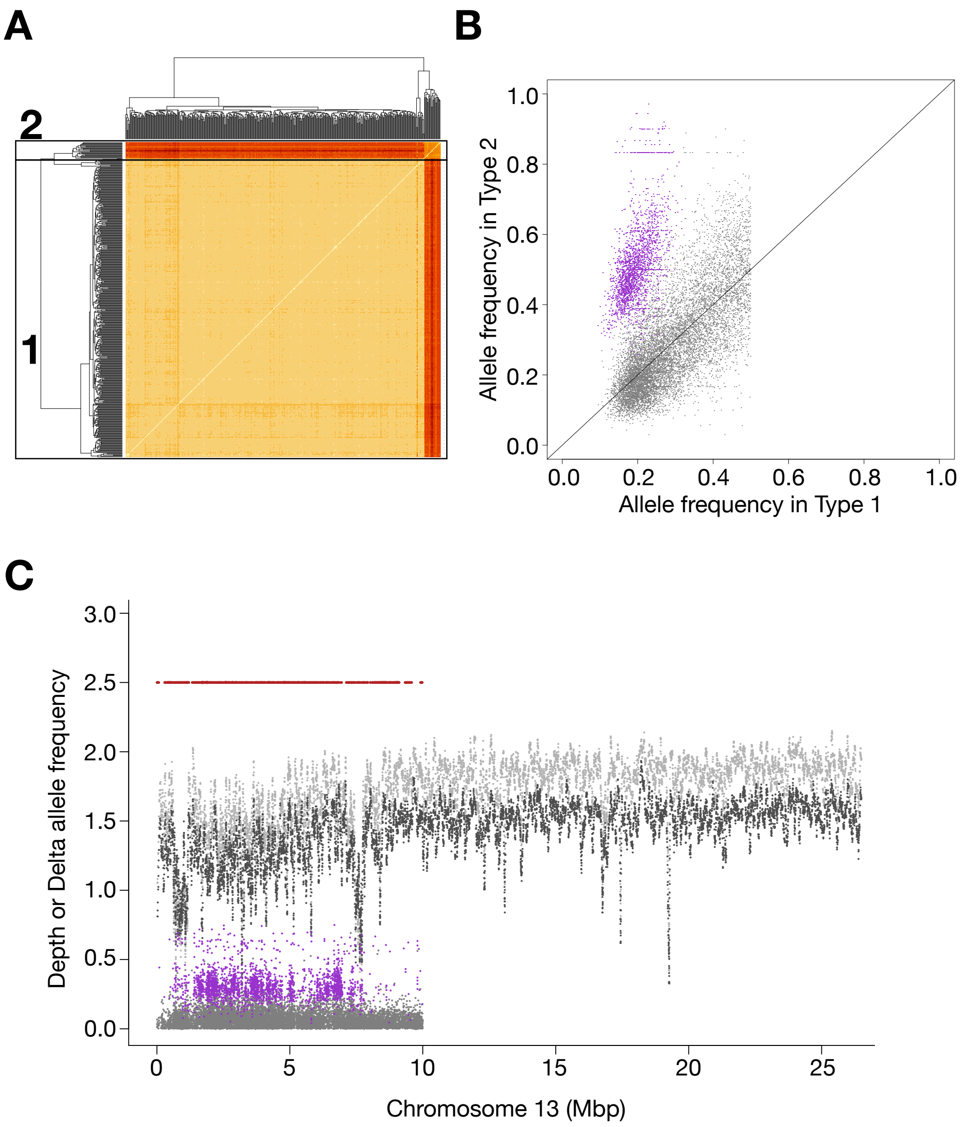

Fig. S3. Genetic variation at chromosome 13:1-7 Mbp. (A) Heatmap where individual *A. anguilla* samples have been clustered based on genotype similarity for >2000 SNPs with high contribution to PC 1 for chromosome 13. (B) Plot of allele frequencies among group 1 individuals on the x-axis vs. group 2 individuals on the y-axis (groupings as indicated in panel A). All frequencies have been transformed to show the minor allele in the Type 1 group. Purple dots represent the SNPs used to construct the heatmap in panel A, grey dots are a set of approximately 10^4 randomly selected background SNPs, and the diagonal line shows equal frequencies in the two groups. As can be seen, the diagnostic SNPs are characterized by being fixed in the majority Type 1 group, while being close to 50% in the Type 2 group suggesting that these are heterozygous at these loci. In contrast, the background loci show very similar allele frequencies in the two groups. (C) Delta allele frequency between groups is shown on the lower left-hand side, with markers colored as in panel B. The dark (group 1) and light (group 2) grey dots are estimated depths of coverage in the two groups. The red line indicates annotated genes over the region containing divergent SNPs. SNPs are colored as in panel B. It is noteworthy that both diagnostic (purple) and background SNPs occur along the entire region showing this signal.

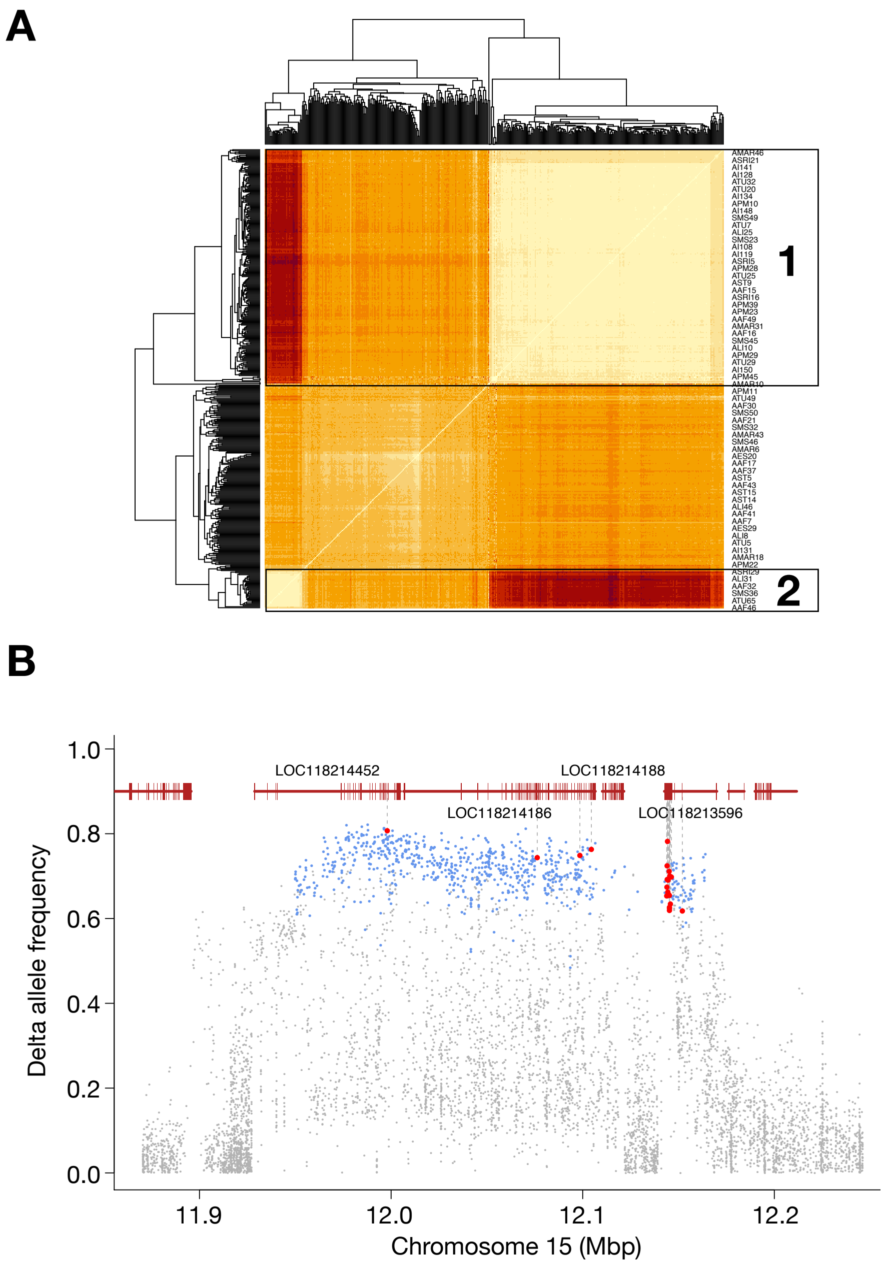

Fig. S4. Genetic polymorphism at chromosome 15:12 Mbp region. (**A**) Heatmap where the individual *A. anguilla* samples have been clustered based on genotype similarity for >700 SNPs with high contribution to PC 1 for chromosome 15. (**B**) Delta allele frequency (DAF) between groups. Blue dots are SNPs used for the heatmap in panel A, and the red dots indicate missense mutations.

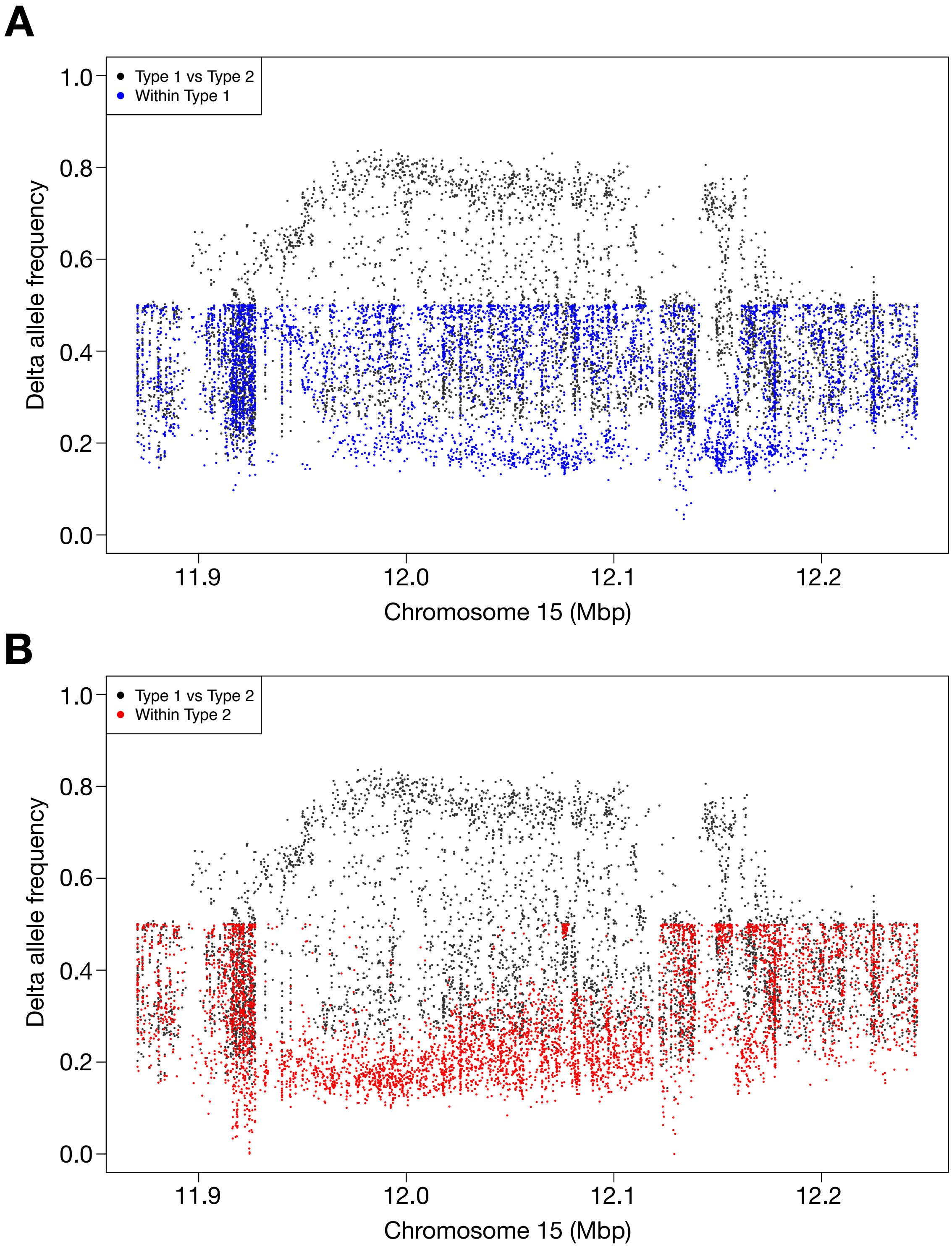

Fig. S5. Characterization of putative inversion on chromosome 15:12 Mbp region. (**A**) Probability of finding different SNP alleles between the two haplotypes in haplotype 1 homozygotes and heterozygotes. (**B**) Same information as in panel A but for haplotype 2.

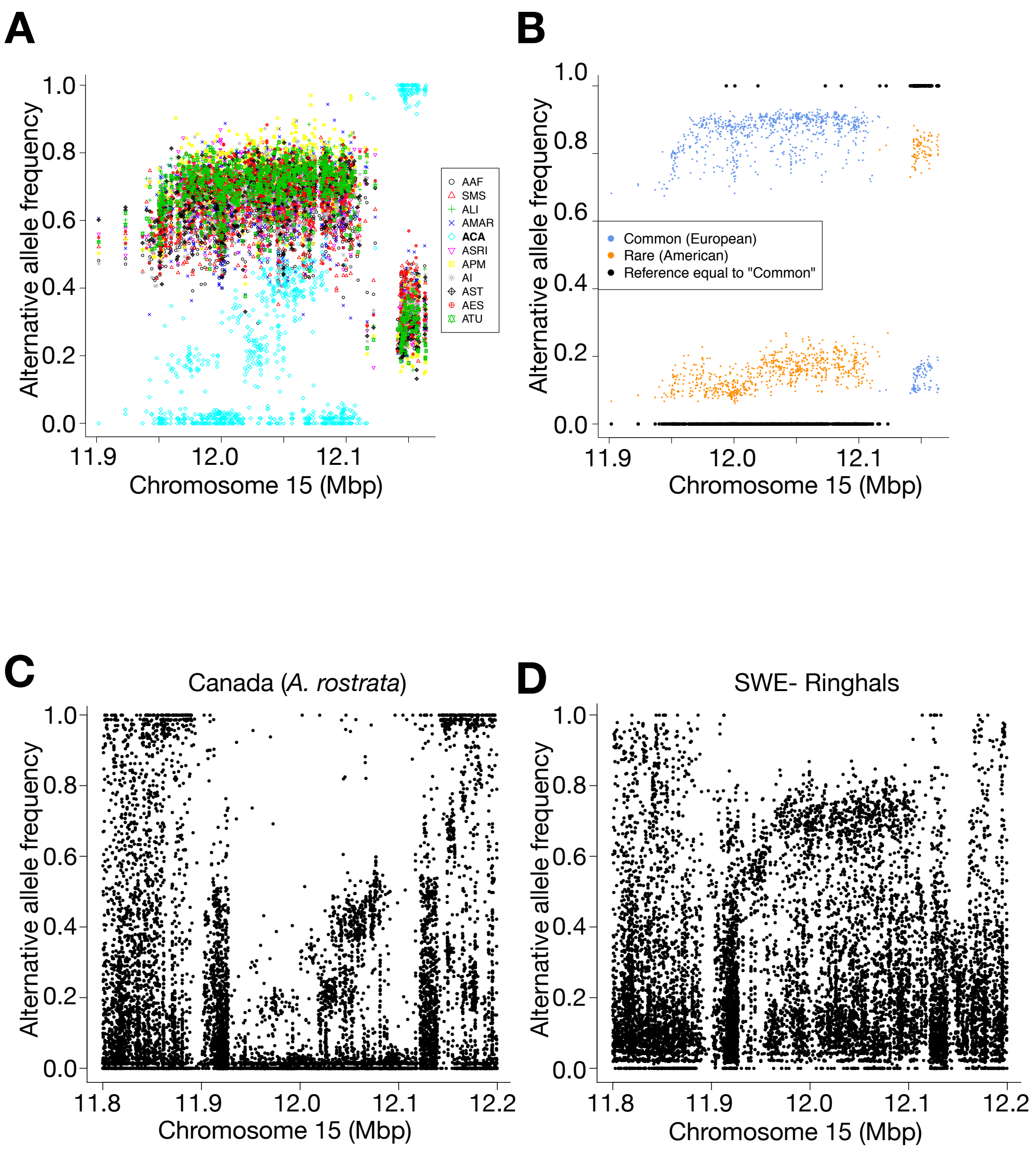

Fig. S6. Further characterization of the putative inversion on chromosome 15:12 Mbp region. (**A**) Alternative (i.e. non-reference) allele frequencies for diagnostic SNPs of all 11 populations. Clearly, all samples are very similar except for the Canadian *A. rostrata* sample (ACA, bolded in the legend, turquoise diamonds); sample abbreviations are as described in Table S1. (**B**) Mean allele frequencies for homozygotes of the two haplotypes (as estimated from individual genotype likelihoods), as well as an indication of whether or not the reference corresponds to the most common (i.e. "European") haplotype or not. (A value of 1 means they are the same, 0 means they differ). The pattern indicates that the reference individual was a heterozygote, and that the assembly has undergone a haplotype-switch in the repeat block located around 12.13 Mbp. Before the switch, the assembly is "American", after it is "European." (**C**) Allele frequencies for all SNPs in the region for the *A. rostrata* eel sample. (**D**) Allele frequencies for all SNPs in the region for the Ringhals samples (Sweden) illustrating higher nucleotide diversity in this region among *A. anguilla* samples.

Table S1. Sample metadata for all eleven localities included in this study

Population Metadata										
#	Country	Location	Date	Short name	Latitude	Longitude	Species	Stage	n	Pairwise θ
1	Canada	Musquash River	1995*	ACA	45.18	-66.25	*rostrata*	Glass	49	0.015
2	England	Severn river	2002*	AES	51.99	-2.17	*anguilla*	Glass	28	0.014
3	France	Arzal	1994*	AAF	47.50	-2.38	*anguilla*	Glass	48	0.014
4	Ireland	River Erne	2001*	AI	54.50	-8.23	*anguilla*	Glass	48	0.014
5	Lithuania	Curonian Lagoon	2001*	ALI	55.54	21.12	*anguilla*	Yellow	48	0.014
6	Morocco	Oued Sebou	1991, 2001*	AMAR	34.27	-6.57	*anguilla*	Glass	50	0.014
7	Portugal	Mira river	1995*	APM	37.72	-8.78	*anguilla*	Glass	48	0.014
8	Sweden	Ringhals	2019	ASRI	57.27	12.11	*anguilla*	Glass	30	0.014
9	Sweden	Strömsån	2019	AST	58.94	11.18	*anguilla*	Yellow	30	0.014
10	Sweden	Motala ström	2001*	SMS	58.59	16.18	*anguilla*	Yellow	48	0.014
11	Tunisia	Oued Medjerda	2002*	ATU	37.01	10.19	*anguilla*	Glass	67	0.014

*Samples previously used in Dannewitz *et al.* (1).

Short name refers to the prefix used in sample names found in Supplementary Figures and SRA accession names. Pairwise nucleotide diversity was calculated per sampling locality using ANGSD and calculating the mean genome-wide value of 5kb windowed estimates.

Supplementary Text

Transcriptome data for chromosome 13. Using previously published transcriptome data from males (2) (https://www.ncbi.nlm.nih.gov/bioproject/PRJNA260119), we called exonic SNPs using STAR-aligner v 2.7.2b (3) followed by haplotype caller in GATK v4.1 (4) within the Sentieon genomic toolset (5). This variant callset was then intersected with our identified genomic SNPs showing an unusual frequency distribution between the two groups of individuals showing genetic differentiation in the chromosome 13 region. Although it has been suggested that eels do not have genetic sex determination (6), we tested the hypothesis that the region of genetic divergence on chromosome 13 is a sex determination region under an X/Y system. In the case of an X/Y system, we would expect all male individuals to be heterozygous at all expressed sites. However, no sample deviated from homozygous reference at more than 25 out of 103 diagnostic exonic sites, and out of 61 unique sites called across all individuals, only 20 were found in more than one individual. These results do not support the observed pattern as being part of a sex-determining mechanism.

Gene content and missense mutations within the putative inversion on chromosome 15:12.1 Mbp region. The putative inversion contains the following genes: *LOC118214452* (FERM, ARHGEF and pleckstrin domain-containing protein 1-like), *LOC118214186* (dedicator of cytokinesis protein 9-like), *LOC118214188* (solute carrier family 15 member 1-like), and *LOC118213596* (G patch domain-containing protein 8-like). Among the SNPs that are strongly differentiated between the two types (DAF > 0.6), there are 21 missense mutations, distributed as follows: 1 in *LOC118214452*, 3 in *LOC118214186* and 17 in *LOC118213596*. It seems plausible that, if there is a fitness effect associated with this segment, it is related to the large number of amino acid changes in *LOC118213596*, where the changes cover most of the protein (from position 320 to1079), while, apparently, maintaining an open reading frame.

Mortality caused by within generation selection. We predicted the mortality required to shift the allele frequencies by 5% at each of 10 independent loci in eels from one geographic region during the life-span of a single generation (cohort), under the assumption of codominance and an initial equal allele frequency (p=0.50). This was calculated using the following relationship:

Genotypes	AA	Aa	aa
Frequency before selection	p^2	2pq	q^2
Relative fitness	1	1-0.5s	1-s
After selection	p^2	2pq(1-0.5s)	$q^2(1-s)$

These calculations show that a selection coefficient of s=0.333 is required to shift the frequency of the A allele from 0.50 to 0.55 at locus 1, resulting in a mortality of 16.5%. The percentage of individuals surviving, after selection has shifted the allele frequencies by 5% at each of 10 independent loci using the same assumptions, can be calculated as $(1-0.165)^{10}=0.16$. Thus, a total mortality of 84% is required to shift the allele frequency by only 5% at each of 10 independent loci within a single generation. A similar shift in allele frequencies at 100 independent loci results in survival rates as low as 10^{-8}.These mortality rates are unrealistically high compared with reported mortality estimates in eel of 99.8% during the trans-Atlantic migration of leptocephali larvae (7) and that the annual mortality rate during the freshwater stage must be

much lower than 10% (8). Furthermore, the present study shows that the relatively high mortality rate during the trans-Atlantic migration has not resulted in striking allele frequency differences between geographic regions since all samples included in the present study are from later stages (glass eels or yellow eels; Table S1).
